# Platelet Count/Spleen Diameter Ratio and Serumv Ascites Albumin Gradient for Screening Parameters of Esophageal Varices in Decompensated Hepatic Cirrhosis: An Observational Study

**DOI:** 10.31729/jnma.v63i292.9263

**Published:** 2025-12-31

**Authors:** Rinku Joshi, Kushal Shrestha, Sunil Shrestha, Sujeeta Bajracharya, Krishti Thapa, Sitaram Khadka

**Affiliations:** 1 Department of Internal Medicine, Shree Birendra Hospital, Nepalese Army Institute of Health Sciences, Kathmandu, Nepal; 2 College of Medicine, Nepalese Army Institute of Health Sciences, Kathmandu, Nepal; 3 Department of Emergency Medicine and General Practice, Shree Birendra Hospital, Nepalese Army Institute of Health Sciences, Kathmandu, Nepal; 4 Department of Anesthesia, Shree Birendra Hospital, Nepalese Army Institute of Health Sciences, Kathmandu, Nepal; 5 Department of Pharmacy, Shree Birendra Hospital, Nepalese Army College of Health Sciences, Kathmandu, Nepal

**Keywords:** *esophageal varices*, *gastrointestinal endoscopy*, *platelet count*, *splenomegaly*, *serum-ascites albumin gradient*

## Abstract

**Introduction:**

In low- and middle-income countries like Nepal, with limited resources, the oesophagogastroduodenoscopy facility may not be available widely due to its high cost and availability constraints. Most of the studies done to predict the presence of esophageal varices in cirrhotic patients are on either serum ascites albumin gradient or platelet count to splenic diameter as the noninvasive parameter. This study aims to assess the platelet count to splenic diameter ratio and serum ascites albumin gradient as non-invasive screening parameters for esophageal varices in patients with decompensated hepatic cirrhosis and ascites.

**Methods:**

An observational study was conducted recruiting decompensated chronic liver disease patients, aged 18 to 65 years, having no previous or recent endoscopic diagnosis of esophageal varices from June to December 2022 using a purposive sampling method. Serum ascites albumin gradient, platelet count, spleen diameter by abdominal ultrasonogram, and platelet count to splenic diameter ratio were calculated. Later upper gastrointestinal endoscopy was performed to confirm the presence of esophageal varices.

**Results:**

Among 150 enrolled patients, 110 (73.33%) had esophageal varices. Of the cases of esophageal varices, 80% had high SAAG, and 68.18% had low platelet count to splenic diameter ratio. SAAG (≥ 1.1 g/dl) alone had a balanced sensitivity of 80% and specificity of 85%.

**Conclusions:**

Serum ascites albumin gradient and platelet count to splenic diameter ratio could be considered as a non-invasive screening parameter of esophageal varices in low-resource settings as a low-cost alternative.

## INTRODUCTION

Esophageal varices (EV) are a common and serious complication of cirrhosis, leading to life-threatening hemorrhage if left undetected.^1^ Although oesophagogastroduodenoscopy is the gold standard for screening, its availability and cost constraints limit its routine use in low- and middle-income settings like Nepal. Non-invasive markers have been proposed for risk stratification of EV. The platelet count to spleen diameter (PC/SD) ratio has emerged as a simple and cost-effective marker for assessing portal hypertension and EV.^2^ Similarly, the serum-ascites albumin gradient (SAAG), traditionally used to assess the etiology of ascites, has been found to predict EV with ascites.^3^

Evaluating the diagnostic performance of PC/SD ratio and SAAG could help optimize screening strategies for EV in resource-constrained settings. This study aims to assess the PC/SD ratio and SAAG as non-invasive screening parameters for EV in patients with decompensated hepatic cirrhosis and ascites.

## METHODS

This hospital-based observational study was conducted in the Internal Medicine Ward of Shree Birendra Hospital over a seven-month period, from June 2022 to December 2022. Prior to commencing data collection, ethical approval was obtained from the Institutional Review Committee of the Nepalese Army Platelet Count/Spleen Diameter Ratio and SerumAscites Albumin Gradient for Screening Parameters of Esophageal Varices in Decompensated Institute of Health Sciences (IRC-NAIHS registration number: 351). A total of 150 patients aged 18 to 65 years with a confirmed diagnosis of chronic liver disease (CLD) who presented with clinical evidence of decompensation were enrolled. Participants were recruited through a non-probability purposive sampling method from among patients admitted to the medical ward during the study period.

To minimize confounding factors, several exclusion criteria were applied. Patients with a history of upper gastrointestinal (UGI) bleeding, those already on non-selective beta-blocker therapy, individuals who had received platelet transfusion within the preceding month, and patients with immune thrombocytopenic purpura were excluded. Since splenomegaly can occur due to various etiologies unrelated to portal hypertension, patients with spleen enlargement secondary to conditions other than cirrhosis were also excluded. Furthermore, individuals older than 65 years were excluded because advanced age is often associated with multiple comorbidities that could independently affect platelet count, splenic size, or portal hemodynamics, thereby acting as potential confounders. Written informed consent was obtained from every participant after explaining the purpose and procedures of the study.

Abdominal ultrasonography was performed for all patients to measure the spleen’s bipolar diameter and evaluate ultrasonographic features of portal hypertension, such as splenomegaly, presence of ascites, and increased portal vein diameter. All ultrasound examinations were carried out by experienced radiologists using standardized protocols to ensure consistency in measurements. The serum-ascites albumin gradient was calculated and categorized into higher (>1.1 g/dL) and lower (<1.1 g/dL) levels according to recommended clinical guidelines.^4^ The platelet count-to-spleen diameter ratio was determined using the cutoff value of 909, originally proposed by Giannini et al.^2^

Oesophagogastroduodenoscopy was subsequently performed for all patients by a gastroenterologist who was blinded to the clinical findings, ultrasonographic data, and laboratory parameters to reduce observer bias. A Pentax Medical endoscope was used for the procedure. Esophageal varices were classified into three grades: Grade 1 (varices that flatten with insufflation or gentle pressure from the endoscope), Grade 2 (non-depressible varices occupying less than one-third of the esophageal lumen), and Grade 3 (large, confluent varices occupying more than one-third of the lumen).^5^ The severity of liver disease was assessed using the Child-Turcotte-Pugh (CTP) scoring system.^6^

Data were compiled and analyzed using the Statistical Package for Social Sciences (SPSS) version 25. Categorical variables, such as demographic characteristics and etiological patterns of CLD, were summarized using frequencies and percentages. Continuous variables, including laboratory parameters and spleen measurements, were expressed as means, standard deviations, and ranges. Diagnostic accuracy measures were calculated by comparing OGD findings with laboratory-based cutoffs. Cases where both OGD and the laboratory parameter (SAAG or PC/SD ratio) indicated the presence of esophageal varices were classified as true positives, whereas cases where both indicated absence were classified as true negatives. Using these classifications, sensitivity, specificity, positive predictive value (PPV), and negative predictive value (NPV) were computed for SAAG and the PC/SD ratio individually, and also for their combined performance.

## RESULTS

Our study included a total of 150 patients who met the inclusion criteria. Among them, 94 (62.67%) were male, and 56 (37.33%) were female. The mean age of the participants was 50.51±11.32 years, with the youngest being 18 years and the oldest 65 years. There were 50 (33.33%) patients who belonged to 46-60 years age group while 48 (32%) cases belonged to 61-55 years age group. There were 78 (52%) cases with chronic live disease related to alcohol consumption while 42 (28%) cases had unknown etiology. Similarly, there were 15 (10%) cases of chronic liver disease related to Hepatits B while there were 3 (2%) cases with Hepatits C (Table 1).

**Table 1:** Demographic characteristics of the patients (n=150).

Variable	n(%)
**Age groups**	
18-30	05(3.33%)
31-45	47(31.33%)
46-60	50(33.33%)
61-65	48(32%)
**Sex**	
Male	94(62.66%)
Female	56(37.33%)
**Etiology**	
Alcohol consumption	78(52%)
Hepatitis B	15(10%)
Alcoholic and Hepatitis B	10(6.67%)
Hepatitis C	3(2%)
Autoimmune	2(1.33%)
Unknown etiology	42(28%)

**Table 2:** Distribution of SAAG and PC/SD according to endoscopy findings (n=150).

Variable	EV (mean±SD)	NEV (mean±SD)
PC (cells per microliter)	106866.88±18229.16	110274.62±19038.32
SD (mm)	116.83 ± 12.86	116.05± 15.03
PC/SD	941.36±267.08	986.33±300.76
SAAG value (g/dl)	2.07 ± 0.80	0.69 ± 0.47

PC = Platelet count, SD = Splenic diameter, SAAG = Serum ascites albumin gradient

**Table 3:** Diagnostic metrics of SAAG and PC/SD (n=150).

Metric	High SAAG (≥11)	Low PC/SD (<909)	Series (both positive)	Parallel (either positive)
Sensitivity	0.8	0.68	0.53	0.95
Specificity	0.85	0.38	0.95	0.28
PPV	0.94	0.75	0.97	0.78
NPV	0.61	0.3	0.42	0.69

PC = Platelet count, SD= Splenic diameter, SAAG = Serum ascites albumin gradient, PPV = Positive predictive value, NPV= Negative Predictive value.

Regarding the severity of liver disease, based on the Child-Turcotte-Pugh classification, 15 patients (10%) were categorized as Class A, 50 patients (33.33%) as Class B, and 85 patients (56.67%) as Class C. Oesophagogastroduodenoscopy demonstrated the presence of esophageal varices (EV) in 110 patients (73.33%). Among these 110 patients, 42 (38.18%) had Grade 1 EV, 54 (49.09%) had Grade 2 EV, and 14 (12.73%) had Grade 3 EV.

The mean SAAG of the study population was 1.7 ± 0.95 g/dL. The SAAG values ranged from a minimum of 0.13 g/dL to a maximum of 2.99 g/dL. The mean platelet count observed was 107,776.61 ± 18,445.9 cells/μL, with values ranging from 83,093 cells/μL to 149,895 cells/μL. Ultrasonographic evaluation showed that the mean splenic bipolar diameter was 116.62 ± 13.42 mm, with the smallest diameter recorded at 90 mm and the largest at 133 mm. Using platelet count and splenic size parameters, the platelet count-to-spleen diameter (PC/SD) ratio was calculated. The mean PC/SD ratio was 953.35 ± 276.15, with values ranging from 637.56 to 1561.41. The distribution of SAAG, platelet count, splenic diameter, and PC/SD ratio among patients with and without EV (Table 2).

Among the 110 patients with EV confirmed by OGD, 88 patients (80%) had a high SAAG (≥ 1.1 g/dL), whereas 22 patients (20%) had a low SAAG (< 1.1 g/dL). Among the 40 patients without EV, 6 patients (15%) had a high SAAG, and 34 patients (85%) had a low SAAG. With respect to the PC/SD ratio, among the 110 EV-positive patients, 75 patients (68.18%) had a low PC/SD ratio (< 909), while 35 patients (31.82%) had a high PC/SD ratio (≥ 909). Among the 40 EV-negative patients, 25 patients (62.5%) had a low PC/SD ratio, and 15 patients (37.5%) had a high PC/SD ratio.

Based on these distributions, diagnostic accuracy measures were calculated. SAAG ≥ 1.1 g/dL demonstrated a sensitivity of 80% and a specificity of 85% for detecting EV. The PC/SD ratio < 909 demonstrated a sensitivity of 68% and a specificity of 38%. When both SAAG and PC/SD ratios were positive, specificity increased to 95% with a corresponding positive predictive value (PPV) of 97%. When either the SAAG or PC/SD ratio was positive, sensitivity increased to 95%, with a corresponding specificity of 28%. The sensitivity, specificity, PPV, NPV, and combined diagnostic values are summarized (Table 3).

## DISCUSSION

Chronic liver disease often complicates with presentation of esophageal varices, which could lead to serious complications in patients requiring emergency medical attention. Endoscopic evaluation is considered the gold standard in the diagnosis of varices, and several guidelines exist for its periodic evaluation when detected. However, the lack of endoscopic facilities at all levels of health care centers, especially in resource-limited settings, should arouse a need for an evidence-based parameter that is more readily available and affordable. This could help reduce the need for invasive procedures like OGD and detect EV before the potential medical complication. The use of SAAG and the PC/SD ratio could be a cost-effective alternative to screening EV in low- and middle-income countries (LMICs).

In our study of 150 patients, SAAG demonstrated a balanced diagnostic performance, with a sensitivity of 80% and specificity of 85%. A substantial proportion of patients (80%) with a high SAAG value had EV confirmed through OGD. This observation is consistent with findings from several international studies. Torres et al in Peru (p=0.028) and Das et al in India (p=0.013) also reported that a high SAAG (>1.1 g/dL) was significantly associated with the presence of EV, reinforcing the value of SAAG as a non-invasive indicator of portal hypertension and variceal risk.^3,7^ These studies collectively suggest that elevated SAAG correlates with increased portal pressure, which in turn predisposes patients to the development of portosystemic collaterals, including esophageal varices.

However, not all studies have reported similar associations. Koirala et al., in a study among 80 cirrhotic patients in Nepal, did not find a statistically significant association between high SAAG (>1.1 g/dL) and the presence of EV. Despite this, their analysis of a higher SAAG threshold (>1.6g/dl) revealed a sensitivity of 78.66% and PPV of 92.18%, comparable to our findings.^8^ This underscores the possibility that SAAG performance may vary depending on disease severity, underlying etiology, sample size, and selected cutoff points. Similarly, Thong et al. demonstrated a significant association between high SAAG and EV (p = 0.0104) and reported that a threshold >1.75 g/dL achieved a sensitivity of 78.4% and specificity of 83.3%.^9^ Eldeeb et al. further highlighted the diagnostic potential of SAAG, noting superior sensitivity (97.4%) and specificity (89.47%) at a cutoff of >1.4 g/dL.^10^ These variations suggest that optimal SAAG thresholds may differ across populations, and context-specific cutoffs may offer improved predictive accuracy.

Conversely, the PC/SD ratio <909 in our study demonstrated suboptimal diagnostic performance, with a sensitivity of 68% and specificity of 38%. These findings are considerably weaker compared to Giannini et al, which suggested near-perfect sensitivity and specificity (93%) for the same cutoff.^2^ A meta-analysis evaluating this ratio across diverse populationsincluding general cirrhotic cohorts and schistosomiasis-related cirrhosisreported pooled sensitivity and specificity of 89% and 74%, respectively, indicating wide variability in its diagnostic utility.^11^ Under alternative threshold settings, performance also varied. In Mexican patients, a cutoff ≤ 884.3 yielded 84% sensitivity and 70% specificity, while in Indian alcoholic cirrhosis, a <997 ratio predicted large EV with 52% sensitivity and 64% specificity.^12^,^13^ These discrepancies highlight the influence of different etiologies, disease stages, and methodological differences on the predictive capacity of hematologic indices.

Our study also found that the mean splenic size was greater in patients with varices (116.83 ± 12.86 mm) compared to those without (116.05 ± 15.03 mm). Although the difference was modest, it aligns directionally with findings from Bhattarai et al., who reported a significantly larger spleen size among patients with varices (153.67 ± 1.21 mm) than those without (126.7 ± 2.35 mm, p < 0.001).^14^ The smaller magnitude in our study may reflect differences in disease chronicity, etiologic distribution, or severity of portal hypertension. Splenomegaly arises due to congestive hypersplenism, and its extent can vary according to both the duration and degree of portal pressure elevation. Additionally, platelet levels in our study were lower among patients with EV, consistent with Gupta et al., who found that platelet count decreased with increasing variceal size (p < 0.001).^15^ This supports the pathophysiological understanding that hypersplenism contributes to thrombocytopenia, which in turn may serve as a surrogate marker for significant portal hypertension.

The limited sample size, a single-center design, and descriptive approach restricted our ability to evaluate formal statistical associations. Multicenter studies with larger cohorts would enhance generalizability and allow for robust comparative analyses. Further, given the operator-dependent nature of ultrasound, interobserver variability remains an important consideration. Future research might incorporate additional non-invasive scoressuch as MELD, APRI, and FIB-4 strengthen predictive accuracy, and combined models could provide a more holistic risk assessment.^13,16^ Cost-analysis comparisons with endoscopy would also clarify the real-world feasibility of integrating these markers into routine clinical practice.

Although the sample size was determined by the study period rather than an a priori calculation, a post-hoc power analysis was performed. For the association between high SAAG (≥1.1 g/dL) and the presence of esophageal varices, the study demonstrated a large effect size (Cohen’s w ≈ 0.58) and very high post-hoc power (~99.9%) at α = 0.05. Only around 24 subjects would have been required to detect this effect with 80% power, indicating that the study was more than adequately powered for evaluating SAAG.

In contrast, the association between the PC/SD ratio (<909) and esophageal varices showed a very small effect size (w ≈ 0.04) and low post-hoc power (~7%). Detecting such a minimal effect with 80% power would require a sample size of over 5,000 patients, suggesting that the lack of statistical significance for PC/SD reflects a genuinely weak and clinically unimportant association, rather than insufficient sample size. This reinforces that PC/SD is unlikely to be a reliable diagnostic marker in this population.

## CONCLUSION

Our study explored the potential utility of SAAG and the PC/SD ratio as low-cost, non-invasive screening tools for identifying esophageal varices in resource-limited settings. Although the study is descriptive in nature, the observed patterns are consistent with findings from larger international studies, suggesting that these parameters may hold practical value where endoscopy is not readily accessible. However, the present study’s sample size and design limit the strength of the conclusions. To determine their true diagnostic applicability and inform clinical guidelines, larger studies with more rigorous methodology are required before these tools can be recommended for widespread implementation.

## Data Availability

The data are available from the corresponding author upon reasonable request.
